# Ten-Year Implant Survival and Functional Outcomes Following Combined Medial Unicompartmental Knee Arthroplasty and Anterior Cruciate Ligament Reconstruction: A Systematic Review and Call for High-Quality Comparative Trials

**DOI:** 10.2106/JBJS.OA.25.00200

**Published:** 2026-01-26

**Authors:** Fardis Vosoughi, Iman Menbari Oskouie, Amir Kasaeian, Masoud Imani, Tianyi David Luo, Abtin Alvand, Pooya Vahedi

**Affiliations:** 1 Department of Orthopedic and Trauma Surgery, Shariati Hospital and School of Medicine, Tehran University of Medical Sciences (TUMS), Tehran, Iran; 2 Urology Research Center, Tehran University of Medical Sciences, Tehran, Iran; 3 Research Center for Chronic Inflammatory Diseases, Tehran University of Medical Sciences, Tehran, Iran; 4 Liver and Pancreatobiliary Diseases Research Center, Digestive Diseases Research Institute, Tehran University of Medical Sciences, Tehran, Iran; 5 Digestive Oncology Research Center, Digestive Diseases Research Institute, Tehran University of Medical Sciences, Tehran, Iran; 6 Department of Biostatistics, School of Public Health, Iran University of Medical Sciences, Tehran, Iran; 7 Indiana Joint Replacement Institute, Fort Wayne, Indiana; 8 Nuffield Department of Orthopaedics (NOC), University of Oxford, Oxford, United Kingdom; 9 Student Research Committee, School of Medicine, Shahid Beheshti University of Medical Sciences, Tehran, Iran

## Abstract

**Background:**

Managing medial compartment knee osteoarthritis (OA) with anterior cruciate ligament (ACL) deficiency, particularly in younger, active patients, remains challenging. Medial unicompartmental knee arthroplasty (UKA) combined with ACL reconstruction (ACLR) (UKACL) has gained interest, yet outcomes remain incompletely defined. This systematic review aims to evaluate the clinical effectiveness, implant survivorship, complications, and patient-reported outcomes after combined UKA and ACLR in end-stage medial OA with ACL deficiency.

**Methods:**

We systematically searched PubMed, Embase, Scopus, and Web of Science. Studies reporting outcomes of UKA performed with ACLR were included; case reports, technical notes, and biomechanical studies were excluded. Risk of bias was assessed with ROBINS-I V2. We extracted clinical outcomes, implant survival, complications, radiographic findings, and validated functional scores.

**Results:**

Fourteen studies comprising 353 patients met the inclusion criteria. Reported survivorship consistently exceeded 90% at 10 years. Ten revisions were reported, most commonly for lateral OA progression. Overall complication rate was 9.06% with no difference between mobile-bearing and fixed-bearing designs. Mobile-bearing implants had a slightly higher bearing dislocation risk, whereas fixed-bearing designs showed marginally higher polyethylene wear. Functional outcomes improved across studies.

**Conclusion:**

Combined UKA and ACLR appears effective for younger, active patients with isolated medial OA and ACL deficiency, yielding high survivorship and consistent functional gains. Given heterogeneity among studies, high-quality, long-term randomized trials are needed to refine patient and implant selection.

**Level of Evidence:**

Level IV, systematic review of nonrandomized studies. See Instructions for Authors for a complete description of levels of evidence.

## Introduction

Anterior cruciate ligament (ACL) injury increases knee osteoarthritis (OA) risk 4-fold to 6-fold^1^. ACL tears predominantly affect active individuals younger than 30 years and predispose to early OA in the 30 to 50 age range with pain, functional impairment, and reduced quality of life^2,3^. Given the high incidence of ACL rupture, clarifying factors that drive postinjury OA is essential^4^.

Unicompartmental knee arthroplasty (UKA) is a well-established and excellent option for end-stage medial OA, with advantages over total knee arthroplasty (TKA)^5^. Optimal management of concurrent medial OA and ACL deficiency remains unsettled^6^. Goodfellow et al. reported worse outcomes and lower survival rates following mobile-bearing UKA in ACL-deficient (ACLD) knees^7^. Traditional contraindications to UKA in the ACLD knee reflect this concern^8^. More recent studies suggest similar results for medial UKA with and without ACL deficiency^8,9^.

Younger high-demand patients present a treatment challenge for knee surgeons^10^. Several therapeutic approaches are suggested for management, including conservative care^11^, HTO alone or with ACLR^12,13^, UKA with ACLR (UKACL)^14^, isolated UKA^15^, and TKA^16^, yet consensus is lacking^6,17^. The link between ACL deficiency and worse outcomes after UKA is primarily reported in studies of mobile-bearing UKAs published over 30 years ago^8^.

UKACL has gained popularity^14,18,19^, and the choice of fixed versus mobile bearings remains debated^20,21^. Considering the potential advantages of this technique and the growing interest in its applications, the purpose of this study was to perform a systematic review of the literature to evaluate the outcomes of combined medial UKA and ACLR in patients with end-stage symptomatic medial compartment OA and ACLD.

## Methods

### Search Strategy

**Fig. 1:**
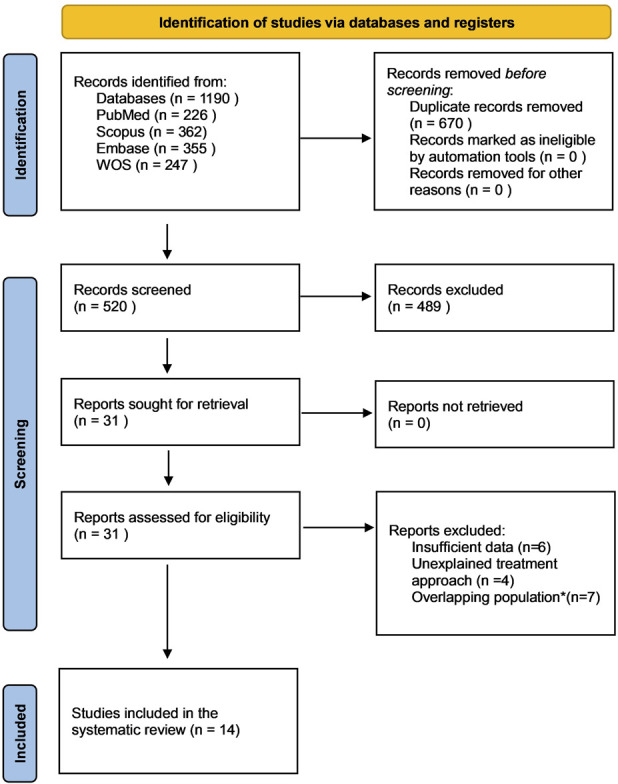
PRISMA flow diagram for the study selection process *Seven studies were excluded due to overlapping patient populations with other included studies from the same institutions and recruitment periods; only one representative study from each overlapping group was retained.

A systematic literature search was conducted across PubMed, Embase, Scopus, and Web of Science from inception to July 2025 in accordance with Preferred Reporting Items for Systematic Reviews and Meta-Analyses (PRISMA) guidelines^22^. (Fig. 1) Search terms combined “unicompartmental knee arthroplasty,” “medial osteoarthritis,” and “ACL,” with database-specific strategies (Supplementary Table I). References of included studies were screened. The protocol was registered at the International Prospective Register Of Systematic Reviews (PROSPERO) (CRD420250652298). The search was conducted primarily by 2 independent reviewers (P.V. and I.M.), followed by full-text screening with disagreements resolved by a senior reviewer (F.V.).

### Data Extraction and Eligibility Criteria

This review included all English and non-English studies published in peer-reviewed journals that reported on UKA performed concomitantly with ACLR in patients with medial knee osteoarthritis and ACL deficiency. Case reports, conference abstracts, symposiums, technical notes, letters, reviews, cadaveric and animal studies, biomechanical studies, and studies with unexplained treatment approaches were excluded. A specified data extraction form was used to extract pertinent details from included articles, encompassing study characteristics, patient data, treatment approaches, and outcome measures. Outcome assessments comprised the following factors:^1^ implantation survival^2^, bearing and graft type^3^, clinical evaluations including all subjective functional scores^4^, reported radiological data, and^5^ complications.

### Risk of Bias (quality) Assessment

Two authors (P.V. and I.M.) independently assessed study quality using the Risk Of Bias In Non-randomized Studies—of Interventions version 2 (ROBINS-I V2) tool developed by Cochrane^23^. Across domains of confounding, intervention classification, participant selection, deviations from intended interventions, missing data, outcome measurement, and selective reporting, disagreements were adjudicated by a third author (F.V.).

### Data Analyses

All statistical analyses were performed using R software (version 4.0; R Foundation for Statistical Computing, Vienna, Austria)^24^. Heterogeneity was evaluated with I^2^ ^25^. Forest plots were generated to visually display the pooled results, while funnel plots evaluated potential publication bias^26^. The difference between mean preoperative and postoperative values was reported as mean differences (MD). Preoperative versus postoperative differences were reported as MD. Survival data were extracted from Kaplan–Meier curves using Plot Digitizer^27^. Data synthesis and analysis, whenever possible, were performed using the “Meta Survival” package in R 4.2.1^28,29^, to construct combined survival curves. Given the non-randomized design, heterogeneity, risk of bias, and methodological variability among the included studies, a meta-analysis was not performed for the overall outcomes.

### Ethics Approval and Consent to Participate

This was a systematic review study using publicly accessible documents as evidence.

## Availability of Data and Materials

The authors confirm that the data supporting the findings of this study are available within the article [and/or] its supplementary materials.

## Result

### Study Selection and Characteristics of the Included Studies

A total of 1,190 records (PubMed 226; Scopus 362; Embase 355; Web of Science 247) articles were identified (Fig. 1). After duplicate removal, 520 records underwent screening, yielding 31 full texts. Ten were excluded (4 with unclear treatment descriptions; 6 with insufficient or unavailable data). This resulted in 21 studies initially eligible for inclusion. Seven studies were found to involve overlapping patients resulting in 14 included studies.

### Quality Assessment and Publication Bias

ROBINS-I V2 assessments are summarized in Fig. 1 of the supplementary files, and funnel plots are shown in Supplementary Fig. 2.

### Participants Characteristics

Across 14 studies, 353 patients were included (Table I). Pain with instability was the most common presentation. All studies focused on isolated ACL deficiency with medial compartment OA; additional ligamentous injuries (posterior cruciate ligament, medial collateral ligament, lateral collateral ligament) were either not reported or listed as exclusion criteria. There was large heterogeneity with respect to study design. The mean age ranged from 46 to 58.9 years and follow-up of 1.7 to 14.6 years. The reported mean BMI ranged from 24.1 to 28.2 kg/m^2^. The mean sample size was 25.2 (range 10-75).

**TABLE I:** Characteristics of the Included Studies

First Author/Publication Year	Study design	Patients (M:F)	Mean age	Age (SD)	Mean Follow-Up	Follow-Up (SD)	Mean BMI
Tang et al., 2024^30^	Retrospective cohort	13 (3:10)	58.9	4.2	7.14	2.45	NR
Jaber et al., 2023^14^	Retrospective case series	23 (18:5)	48	6.25	10	2.12	NR
Aslan and Çevik, 2022^31^	Retrospective case series	12	NR	NR	3.8	0.36	NR
Kurien et al., 2022^32^	Retrospective case series	24 (16:8)	48.8	8.17	5.1	2.87	24.1
Derreveaux et al., 2022^33^	Retrospective cohort	20 (7:13)	58	6.7	2.5	1.41	26.5
Kennedy et al., 2019^34^	Prospective case series	75 (59:16)	52.6	8.75	6.4	3.5	28.2
Iriberri et al., 2019^35^	Retrospective case series	8 (5:3)	52	4.5	14.6	2.93	NR
Tecame et al., 2019^36^	Retrospective case series	24 (20:4)	Mobile: 47.8	Mobile: 3	Mobile:4.41	0.69	NR
			Fixed: 48.4	Fixed: 2.75	Fixed:3.5	0.55	
Ventura et al., 2017^37^	Retrospective case series	14 (9:5)	55	3.5	2.22	0.33	NR
Tian et al., 2015^38^	Prospective Case Series	32 (12:20)	50	NR	4.58	2.92	NR
Weston-Simons et al., 2012^39^	Prospective case series	51 (40:11)	51	7.75	5	0.18	NR
Tinius et al., 2007^40^	Prospective Case Series	32 (25:7)	46	4.25	2.58	0.58	NR
Dervin et al., 2007^21^	Prospective case series	10 (5:5)	52	6	1.7	0.72	NR
Pandit et al., 2006^41^	Prospective cohort	15 (13:2)	49.8	6	2.8	0.45	NR

F = female, M = male, NR = not reported, and SD = standard deviation.

Age and follow-up values are reported in years.

### Implant Survival

**Fig. 2:**
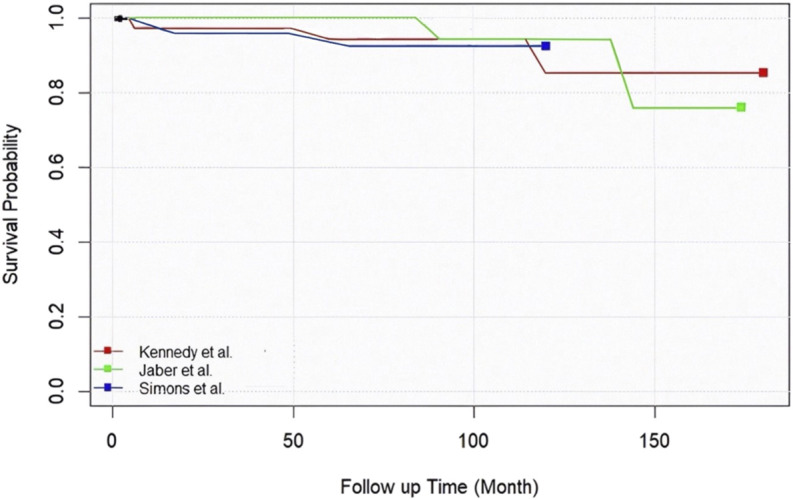
This figure shows the survival curves extracted from the included studies. The red line represents the data from Kennedy et al., the blue line corresponds to Weston-Simons et al., and the green line depicts the findings by Jaber et al.

Three studies reported survivorship explicitly. Jaber et al.^14^ observed 100% at 5 years, 95.7% at 10 years, and 91.4% at 14.5 years. Kennedy et al.^34^ reported 97.0% at 5 years, 92.3% at 10, and 15 years. Weston-Simons et al.^39^ reported 92.7% at 5 and 8 years (Fig. 2). Of the remaining studies, 3 reported revision counts^33,35,41^. Among the 6 studies with revision data^14,33-35,39,41^, the 5-year cumulative revision rate was 3.7% (6 revisions among 161 patients), corresponding to a 5-year survival of 96.3%. Studies with less than 2 years of follow-up reported early function but were insufficient to address durability.

### Complications

Ten UKA revisions occurred among 353 cases (Table II). Revision indications were lateral OA progression (5, 50%) ^14,34,39^periprosthetic joint infection (3 cases, 30%)^34,39,41^, and aseptic loosening (2, 20%)^33,35^. Reported nonrevision complications included 3 bearing dislocations (mobile-bearing)^38,39^, 2 arthroscopic releases^21,40^, 1 lateral meniscus injury^35^, 1 thrombosis^40^, 9 hemarthroses^40^, and 1 arthroscopic loose-body removal^37^. Five graft failures were reported in one study^32^. Additional revisions noted by Legnani et al.^19^ and Ventura et al.^42^ were not duplicated in our table due to cohort overlap with Ventura et al^37^. Foissey et al.^43^ reported 2 arthroscopic arthrolyses for stiffness, excluded from the complication table to avoid duplication with Derreveaux et al^33^.

**TABLE II:** Complications and Revisions

First Author/Publication Year	Follow-up/Last Follow-up (Year)	Total Patients Revision	UKA/ACL Revisions	UKA Revision Rate (%)	Implant Survival (%)	Cause of Revision	Year of Revision	Other Complications
Tang et al., 2024^30^	Mean 7.14/11	13	0/0	0	100	None	None	None
Jaber et al., 2023^14^	Average 10/14.5	23	2/0	8.69	91.30	Trauma & lateral POA	6 and 12 years postoperatively	None
Aslan and Çevik 2022^31^	Mean 3.8/4.33	12	0/0	0	100	None	None	None
Kurien et al., 2022^32^	Mean 5.1/12.8	24	0/5	0	79.2	None	None	1 patient had a failed Gortex ACL graft, 1 had a failed Carbon fiber graft, and the remaining 3 had failed hamstring grafts at 28 months post-UKR
Derreveaux et al., 2022^33^	Mean 2.5/5.09	20	1/0	5	95	aseptic loosening	at 5 years follow-up	None
Kennedy et al., 2019^34^	Mean 6.4/15	75	3/0	4	96	2 lateral POA/1 PJI	one after 5 months/1 after 4 years/1 after 9.9 years	None
Iriberri et al., 2019^35^	Mean14.6/21.5	8	1/0	12.5	87.5	aseptic loosening	after 117 months	1 lateral meniscus tear repair
Tecame et al., 2019/M group^36^	Mean 4.41/NR	9	0/0	0	100	None	None	None
Tecame et al., 2019/F group^36^	Mean 3.5 years/NR	15	0/0	0%	100%	None	None	None
Ventura et al., 2017^37^	Mean 2.22/3.33	14	0/0	0%	100	None	None	1 arthroscopic loose body removal (16 months post-op)
Tian et al., 2015^38^	Mean 4.58/9.3	32	0/0	0	100	None	None	2 Mobile bearing dislocation (exchanged)
Weston-Simons et al., 2012^39^	Mean 5/10	51	2/0	3.84	96.15	1 PJI/1 lateral POA	NR	1 bearing dislocation (exchanged)
Tinius et al., 2007^40^	Mean 2.58/3.16	32	0/0	0	100	None	None	9 hemarthrosis (aspirated), 1 thrombosis in the second postoperative year, 1 arthroscopic release
Dervin et al., 2007^21^	Median 1.7/9	10	0/0	0	100	None	None	1 arthroscopic release
Pandit et al., 2006^41^	Mean 2.8/4.3	15	1/0	6.66	93.33	1 PJI	NR	None

ACL = anterior cruciate ligament, and UKA = unicompartmental knee arthroplasty.

### Surgical Techniques

Six studies used a fixed-bearing configuration, 7 used mobile-bearing, and 1 incorporated both. Mean operative time, reported in 6 studies, ranged from 88.5 to 105 minutes^21,30,37,40^. Most prostheses were cemented; 1 study included cemented, cementless, and hybrid fixation^31^, and 2 did not specify cementation^21,34^.

ACLR was most commonly used hamstring autograft (11 studies)^14,21,30,31,33-38,40^; 2 studies used hamstring or bone–patellar tendon–bone autograft^39,41^, and 1 used allograft^32^.). Eight studies performed single-stage UKACL^14,30-32,35-38^, 4 used staged procedures^21,33,39,40^; 2 studies tailored the choice of staged versus single-stage surgery to patient-specific clinical factors^34,41^.

Common implant systems were Smith & Nephew (7 studies)^21,33,35-38,41^, Zimmer (4)^30-32,34^, and Oxford (2)^14,39^. Surgical sequencing varied across reports: some surgeons performed ACLR after UKA, others started with ACLR, and several used a combined or overlapping approach. In 7 studies, the tibial tunnel guide angle was 55°^21,30,31,35-38^. Intra-articular tunnel position was described as slightly lateral in 3 studies^31,36,37^ and anterior in 2 studies^14,33^. Graft fixation most commonly used a femoral Endo Button with tibial interference screw (Table III).

**TABLE III:** Surgical Techniques

First Author/Publication Year	Bearing Type	Graft Type	Cementation	Staged	Guide Angle	Type of Prosthesis	Mean Surgery Duration (Min)
Tang et al., 2024^30^	Fixed	Hamstring	Cemented	Single	55	Zimmer Biomet	88.5
Jaber et al., 2023^14^	Mobile	Hamstrings	Cemented	Single	NR	Oxford UKA	NR
Aslan and Çevik, 2022^31^	Mobile	Hamstrings	Cementless (5)	Single	55	Zimmer	NR
			Cemented (4)				
			Hybrid (3)				
Kurien et al., 2022^32^	Fixed	Hamstrings	Cemented	Single	NR	Physica ZUK (Lima Corporate, Udine, Italy)	NR
		BPTB					
		Allograft					
Derreveaux et al., 2022^33^	Fixed	Hamstrings	Cemented	11 single	NR	Journey II Uni (Smith and Nephew) in 26 cases	NR
				9 staged		HLS Uni evolution (Tornier) in 8 cases	
Kennedy et al., 2019^34^	Mobile	Hamstrings	Cementless (NR)	Single or Staged, based on the complaint	NR	Zimmer Biomet	NR
			Cemented (NR)				
Iriberri et al., 2019^35^	Fixed	Hamstrings	Cemented	Single	55	Smith & Nephew	NR
Tecame et al., 2019^36^	Fixed: 15	Hamstrings	Cemented	Single	55	Smith & Nephew	NR
	Mobile: 9						
Ventura et al., 2017^37^	Fixed	Hamstrings	Cemented	Single	55	Smith & Nephew	98
Tian et al., 2015^38^	Mobile	Hamstrings	Cemented	Single	55	Smith & Nephew	NR
Weston-Simons et al., 2012^39^	Mobile	BPTB (?)	Cemented	33 single	NR	Oxford UKA	NR
		Hamstring (?)		18 staged			
Tinius et al., 2007^40^	Fixed	Hamstrings	Cemented	21 single	NR	Repicci-II; Eius	95
				11 staged			
Dervin et al., 2007^21^	Mobile	Hamstrings	NR	9 single	55	Smith & Nephew	105
				1 staged			
Pandit et al., 2006^41^	Mobile	Hamstring (11)	Cemented	4 patients simultaneously	NR	Smith & Nephew	NR
		3 BPTB (3)		11 patients had ACL reconstruction first, and arthroplasty later if needed			

BPTB = bone patellar tendone bone autograft, NR = not reported, SD = standard deviation, and UKA = unicompartmental knee arthroplasty.

### Rehabilitation

Most rehabilitation protocols were brace-free with immediate or day 1 knee motion. Early goals emphasized full extension and progressive flexion; isometric quadriceps exercises began within 6 to 24 hours. Partial weight-bearing from day 1 typically continued for 4 to 6 weeks. Some protocols limited flexion to 90° initially, then advanced as tolerated. Proprioceptive training often began around week 4. Programs were generally UKA-based with adjustments for return to sport; continuous passive motion and pain-adapted physiotherapy were variably used.

### Radiographic Outcomes

Radiographic data showed changes in knee alignment and stability. Tang et al.^30^ noted hip-knee ankle angle improvement from 174.07° ± 2.06 to 177.79° ± 1.25 and a posterior tibial slope (PTS) decrease from 7.93° ± 1.07 to 4.57° ± 0.94. Tecame et al.^36^ likewise observed decreases in PTS and varus angles with both bearing types. Aslan et al.^31^ reported varus reduction from 3.6° ± 1° to 2.6° ± 1°, while Tian et al. ^38^reported maintained varus and valgus angles and posterior tibial prosthesis tilt at the last follow-up. Derreveaux^33^ described preoperative mechanical femorotibial angles averaging 178° ± 6°.

### Functional Outcomes and PROMs

Validated PROMs included the American Knee Society Score (AKSS; objective and functional), the Oxford Knee Score (OKS), the Knee Society Score (KSS; knee and function scores), the Tegner Activity Scale, and the Western Ontario and McMaster Universities Osteoarthritis Index. Three studies (80 patients) reported objective AKSS improvements (MD 32-44; I^2^ = 33.4%). The functional AKSS component improved by 10 to 13 points across 3 studies (80 patients) with no heterogeneity (I^2^ = 0.0%). For OKS, 3 fixed-bearing studies (51 patients) reported MD 14.2 to 26; 5 mobile-bearing studies (185 patients) reported MD 12 to 19.1. Three studies (84 patients) reported KSS improvements: knee score MD 32.8 to 54.2 (I^2^ = 93.6%) and function score MD 28.8 to 48.6 (I^2^ = 95%). Tegner improved by 0.8 to 2.2 points across 7 studies (234 patients) with high heterogeneity (I^2^ = 80.4%) (Supplementary Fig. 3).

Beyond PROMs, multiple studies showed significant functional improvements across KOOS subscales (except Sports), OKS, EQ-5D, EQ-VAS, Lysholm, UCLA, IKDC-2000, Tegner activity, and VAS pain ^14,31^. Kurien et al. also reported improved knee stability^32^. Objective testing supported restored ligament function: Tecame et al. found negative Lachman and anterior drawer signs^36^, and Ventura et al. observed clinically acceptable KT-1000 side-to-side differences^37^. Overall, UKACL improved pain and function while restoring objective stability (Supplementary Table II).

## Discussion

This systematic review evaluated medial UKA performed in conjunction with ACLR (UKACL), assessing implant survivorship, functional, radiological, and patient-reported outcomes. The main finding of this study was that UKACL is associated with implant survival consistently exceeding 90% at 10 years in appropriately selected patients. Kennedy et al. and Jaber et al. reported 10-year survivorship of 92.3% and 95.7%, respectively^14,34^, and Weston-Simons et al. reported 92.7% at 5 to 8 years^39^. Across 6 studies, the 5-year cumulative revision rate was 3.7% (5-year survival 96.3%). These results are comparable with those of isolated UKA in ACL–intact (ACLI) knees (5- and 10-year survival, 90.5% and 83.5%, respectively)^44^, challenging the presumption that ACL deficiency necessitates TKR^8,45^.

Historically, ACL deficiency was considered a contraindication to UKA^8^ due to concerns over increased anteroposterior and rotational instability leading to early implant failure. Fixed-bearing designs may mitigate sagittal laxity, but rotational laxity persists^46^. As noted by Zumbrunn et al.^46^, isolated UKA in ACLD patients—especially older, low-demand individuals—may be successful only in the presence of clinical rotational stability. However, this conclusion derives from an elderly cohort (mean age >60 years), creating a selection bias in the existing literature.

By contrast, all 14 UKACL studies in this review reported mean ages <60 years. Mancuso et al.^6^ found superior midterm survival with UKACL (97%) versus UKA in untreated ACLD knees (88%), noting that ACLD-UKA cohorts were older (>60 years) while UKACL cohorts were younger (<55 years). Du et al. reported similar outcomes for ACLD-UKA and ACLI-UKA in 8 studies; however, all included populations had mean ages older than 60 years^9^. Thus, the literature supports ACLD-UKA primarily in older patients, whereas the current review supports UKACL in younger individuals. Potential explanations include stabilizing effects of osteophytes and advanced OA in the elderly and better tolerance of ACL deficiency in low-demand populations.

This study shows consistent improvements across several PROMs (AKSS, KSS, Tegner). Despite variability in reporting functional scores, meaningful conclusions about PROMs remain evident. Eight studies showed consistent OKS improvements of 12 to 26 points^30-32,34,37-39,41^, and 7 studies reported Tegner gains of 0.8 to 2.2 points^14,32,34,37-39,41^ (Supplementary Fig. 3). These results align with prior literature and highlight the clinical value of restoring ligamentous stability in these patients.

Regarding implant design, although biomechanical studies suggest mobile-bearing UKA may better replicate normal kinematics and reduce wear^20,47^, our review did not find consistent superiority of mobile-bearing implants in terms of clinical outcomes. This suggests that in the setting of UKACL, the theoretical biomechanical advantages of mobile bearings may not translate into clinically meaningful gains, possibly due to the remaining laxity after ACLR. Collectively, current evidence supports UKACL as a viable option for younger, active patients with isolated medial OA and ACL deficiency^34^, with several studies reporting excellent subjective and objective outcomes up to 8 to 10 years^19,42,48-50^. Kennedy et al.^34^ also reported low revision and conversion rates with both staged and simultaneous procedures (Supplementary Fig. 4).

Despite promising findings, the current review has serious limitations. Heterogeneity in surgical techniques, implant designs, and patient selection criteria hindered meta-analytic synthesis. Publication bias remains a concern despite comprehensive searching. Long-term, adequately powered randomized controlled trials comparing UKACL to isolated UKA are needed. Future work should incorporate preoperative stability, age and activity level, implant design, and radiographic alignment into predictive models to refine indications and optimize outcomes.

## Conclusion

Combined medial UKA and ACLR is associated with sustained functional improvement (AKSS, OKS, KSS, Tegner) and exceeded 90% 10-year survivorship in appropriately selected patients. For younger, active individuals with isolated medial OA and ACL deficiency, UKACL represents a viable surgical option. Further high-quality prospective studies, including randomized trials, are warranted to establish optimal patient selection criteria, refine surgical technique, and benchmark outcomes against alternative strategies.

## Funding

The research presented in this manuscript received no external form of funding.

## Appendix

Supporting material provided by the authors is posted with the online version of this article as a data supplement at jbjs.org (http://links.lww.com/JBJSOA/B93). This content was not copyedited or verified by JBJS.
