# Erratum: Ten-Year Implant Survival and Functional Outcomes Following Combined Medial Unicompartmental Knee Arthroplasty and Anterior Cruciate Ligament Reconstruction: A Systematic Review and Call for High-Quality Comparative Trials

**DOI:** 10.2106/JBJS.OA.ER.25.00200

**Published:** 2026-02-17

**Authors:** Fardis Vosoughi, Iman Menbari Oskouie, Amir Kasaeian, Masoud Imani, Tianyi David Luo, Abtin Alvand, Pooya Vahedi

In the article by Vosoughi et al., appearing in *JBJS Open Access*, Vol. 11, No. 1, e25.00200, entitled “Ten-Year Implant Survival and Functional Outcomes Following Combined Medial Unicompartmental Knee Arthroplasty and Anterior Cruciate Ligament Reconstruction: A Systematic Review and Call for High-Quality Comparative Trials,” there were errors.

Specifically, the author Amir Kasaeian’s affiliation was “Clinical Research Development Unit, Shariati Hospital, Tehran University of Medical Sciences, Tehran, Iran” but should have been “Research Center for Chronic Inflammatory Diseases, Tehran University of Medical Sciences, Tehran, Iran; Liver and Pancreatobiliary Diseases Research Center, Digestive Diseases Research Institute, Tehran University of Medical Sciences, Tehran, Iran; and Digestive Oncology Research Center, Digestive Diseases Research Institute, Tehran University of Medical Sciences, Tehran, Iran.”

The errors have been corrected in the article online. The authors regret their oversight.
